# Sarcoidosis-associated pulmonary hypertension due to pulmonary arteries stenosis – a case report

**DOI:** 10.1186/s12890-024-03152-0

**Published:** 2024-07-16

**Authors:** Malgorzata Sobiecka, Izabela Siemion-Szczesniak, Barbara Burakowska, Marcin Kurzyna, Malgorzata Dybowska, Witold Tomkowski, Monika Szturmowicz

**Affiliations:** 1 1 st Department of Lung Diseases, National Tuberculosis and Lung Diseases Research Institute, Plocka 26, Warsaw, 01-138 Poland; 2 Department of Radiology, National Tuberculosis and Lung Diseases Research Institute, Plocka 26, Warsaw, 01-138 Poland; 3 Department of Pulmonary Circulation, Thromboembolic Diseases and Cardiology European Health Center Otwock, Medical Centre for Postgraduate Education, Otwock, Poland

**Keywords:** Sarcoidosis, Pulmonary hypertension, SAPH, Treatment, Corticosteroids, Balloon pulmonary angioplasty

## Abstract

**Background:**

Sarcoidosis-associated pulmonary hypertension (SAPH) is listed in Group 5 of the clinical classification of pulmonary hypertension, due to its complex and multifactorial pathophysiology. The most common cause of SAPH development is advanced lung fibrosis with the associated destruction of the vascular bed, and/or alveolar hypoxia. However, a substantial proportion of SAPH patients (up to 30%) do not have significant fibrosis on chest imaging. In such cases, the development of pulmonary hypertension may be due to the lesions directly affecting the pulmonary vasculature, such as granulomatous angiitis, pulmonary veno-occlusive disease, chronic thromboembolism or external compression of vessels by enlarged lymph nodes. Based on the case of a 69-year-old female who developed SAPH due to pulmonary arteries stenosis, diagnostic difficulties and therapeutic management are discussed.

**Case presentation:**

The patient, non-smoking female, diagnosed with stage II sarcoidosis twelve years earlier, presented with progressive dyspnoea on exertion, dry cough, minor haemoptysis and increasing oedema of the lower limbs. Computed tomography pulmonary angiography (CTPA) showed complete occlusion of the right upper lobe artery and narrowing of the left lower lobe artery, with post-stenotic dilatation of the arteries of the basal segments. The vascular pathology was caused by adjacent, enlarged lymph nodes with calcifications and fibrotic tissue surrounding the vessels. Pulmonary artery thrombi were not found. The patient was treated with systemic corticosteroid therapy and subsequently with balloon pulmonary angioplasty. Partial improvement in clinical status and hemodynamic parameters has been achieved.

**Conclusions:**

An appropriate screening strategy is required for early detection of pulmonary hypertension in sarcoidosis patients. Once SAPH diagnosis is confirmed, it is crucial to determine the appropriate phenotype of pulmonary hypertension and provide the most effective treatment plan. Although determining SAPH phenotype is challenging, one should remember about the possibility of pulmonary arteries occlusion.

## Background

Sarcoidosis is a multisystem inflammatory granulomatous disorder of unknown aetiology, most commonly affecting the lungs and intra-thoracic lymph nodes. However, the disease can also involve other organs and systems, such as the heart, kidneys, musculoskeletal system, brain, eyes, skin, and liver [1–4]. In most patients with pulmonary sarcoidosis, the course of the disease is favourable with complete or partial remission of the lesions, often without systemic treatment. However, some patients develop progressive pulmonary fibrosis and/or pulmonary hypertension (PH), associated with significant morbidity and increased mortality [5, 6]. The prevalence of sarcoidosis-associated pulmonary hypertension (SAPH) ranges from 3 to 20% in sarcoidosis patients referred to the tertiary centres, and reaches up to 74% in the population with advanced disease, listed for lung transplantation [7–11]. The pathophysiological mechanisms of SAPH are complex and multifactorial, and thus several causes may coexist in the same patient [12–14]. According to recently published European Society of Cardiology/European Respiratory Society (ESC/ERS) guidelines, SAPH is listed in Group 5 of the clinical classification, as pulmonary hypertension with unclear and/or multifactorial mechanisms [15].

The most common cause of SAPH development is lung fibrosis with the associated destruction of the vascular bed, and/or chronic alveolar hypoxia [13, 16, 17]. However, SAPH may occur even in the absence of significant lung disease or in the patients with near-normal lung function indices, that is suggestive of other pathogenic mechanisms. Postcapillary PH due to cardiac sarcoidosis or PH associated with other comorbidities (portopulmonary hypertension, obstructive sleep apnoea) have to be considered as the causes of SAPH [13, 18, 19].

Direct vascular involvement in SAPH patients is seen rarely, and it may present with occlusion of pulmonary arteries mostly due to coexisting venous thromboembolic disease, or external obstruction of large pulmonary arteries caused by markedly enlarged mediastinal/hilar lymph nodes, and/or with fibrotic tissue localized in hilar/mediastinal region [13, 14, 20–23]. Complete occlusion of pulmonary arteries is extremely rare, nevertheless it has to be considered in the differential diagnosis of severe PH in the course of sarcoidosis.

## Case presentation

A 69-year-old, non-smoking female, with arterial hypertension, hyperlipidaemia and nephrolithiasis, diagnosed with stage II sarcoidosis twelve years earlier, was admitted to the Department of Lung Diseases due to progressive dyspnoea on exertion, dry cough, minor haemoptysis and increasing oedema of the lower limbs. The diagnosis of sarcoidosis was confirmed by histopathological examination of two mediastinal lymph nodes obtained by mediastinoscopy. The examined lymph nodes contained well-formed non-necrotizing granulomas built with epithelioid and giant cells. Staining and cultures for acid-fast bacilli and fungi were negative. At the beginning, the course of the disease was stable and she did not require immunosuppressive therapy. Three years before the admission to our department, the patient noticed deterioration in exercise tolerance and was seen at the pulmonology outpatient clinic. Pulmonary function tests showed moderate obstruction (forced expiratory volume in 1 s; FEV_1_ 1,34 l; 62% of the predicted value, forced vital capacity; FVC 2,29 l; 88% of the predicted value, FEV_1_/FVC 59%) and mild decrease in lung transfer factor for carbon monoxide (TLCO; 4,12 mmol/min/kPa; 56% of the predicted value). Non-contrast chest computed tomography (CT) revealed progression of hilar and mediastinal lymphadenopathy, and of nodular lung disease. Additionally, narrowing of the middle lobe bronchus and of the bronchus to the third left lung segment, were described. The patient was treated with an inhaled long acting beta-mimetic and an inhaled glucocorticosteroid with clinical improvement. Due to the COVID-19 pandemic, visits to the pulmonology outpatient clinic were irregular. Considering the progression of lung disease and increasing clinical symptoms, the patient was referred to the hospital.

On admission, the physical examination revealed an underweight patient (BMI 17.5 kg/m^2^) with resting dyspnoea, oedema of the lower limbs, dullness to percussion and reduced breath sounds over the left lower lung field. The patient was in World Health Organization (WHO) functional class IV. Laboratory tests showed normal WBC with lymphopenia (0.77 × 10^9^/L, normal range 1.18–3.74 × 10^9^/L), elevated concentrations of D-Dimer (958 ng/ml, normal < 500 ng/ml) and plasma natriuretic peptide (NT-pro BNP) (1377.0 pg/ml, normal range 0.0-125.0 pg/ml). Blood gas analysis presented hypoxemia and hypocapnia (pO_2_ – 53.6 mmHg, PaCO_2_ – 29.4, pH – 7.48). Calcium concentrations in serum and in daily urine collections were within normal limits. Bedside transthoracic echocardiography, performed on admission, revealed signs indicating a high probability of pulmonary hypertension. The right ventricle (RV) was dilated - in four-chamber view the diameter of the right ventricle was 47 mm vs. the diameter of the left ventricle (LV) 35 mm (RV/LV ratio 1.3). The pulmonary artery was also dilated to 34 mm. Severe tricuspid valve regurgitation was diagnosed with an increase in the peak tricuspid valve regurgitation velocity (TRV) to 5.8 m/s. The estimated systolic pulmonary artery pressure (sPAP) was approximately 140 mmHg and the intraventricular septum was distinctly flattened. Tricuspid annular plane systolic excursion (TAPSE) measured with M-Mode was decreased to 17 mm. A small pericardial effusion was found. Preserved morphology and function of LV was observed, with LV ejection fraction above 50%.

**Fig. 1:**
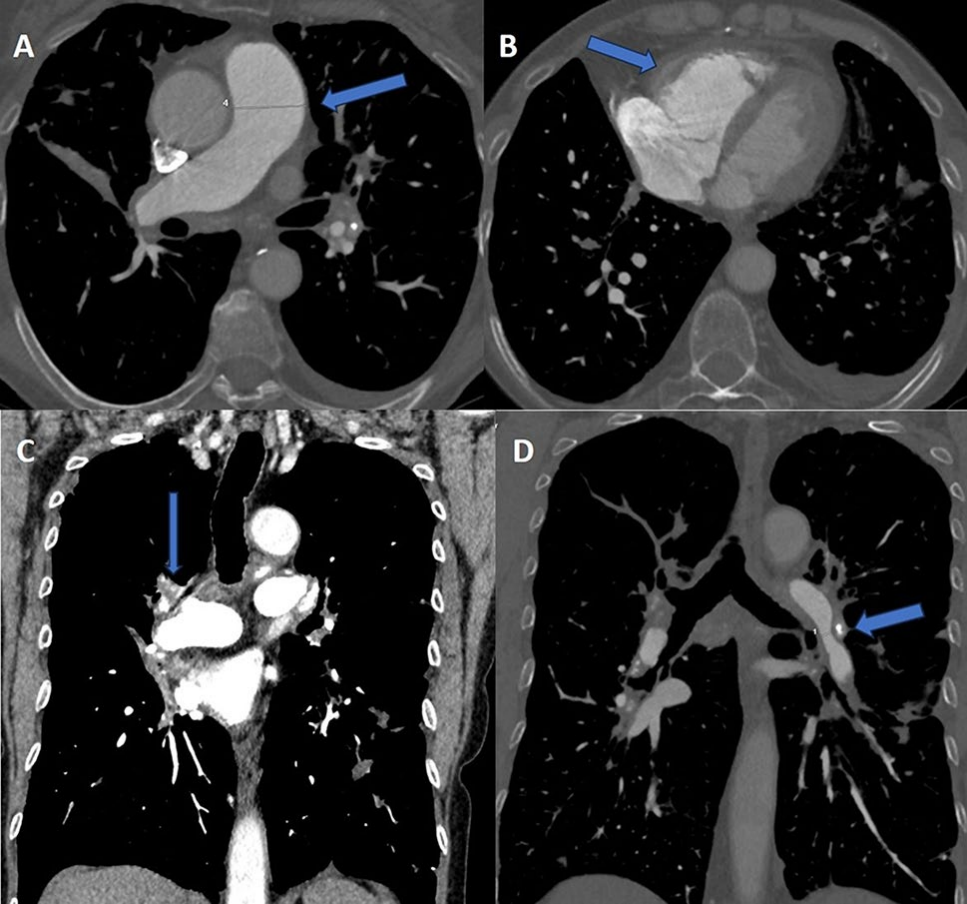
Computed tomography pulmonary angiography (CTPA) a 69-year-old-female with sarcoidosis: (**a**) axial image, mediastinum window, at the level of the pulmonary trunk shows a dilatation of the pulmonary trunk to 33 mm (blue arrow); (**b**) axial image at the level of the heart shows enlarged right ventricle (blue arrow) and mild pleural effusion on the left side; (**c**) coronal image demonstrates complete occlusion of the right upper lobe artery (blue arrow); (**d**) coronal image reveals stenosis of the left lower lobe artery just below the branch of the artery to segment six with post-stenotic dilatation, and enlarged calcified lymph nodes on this level (blue arrow)

**Fig. 2:**
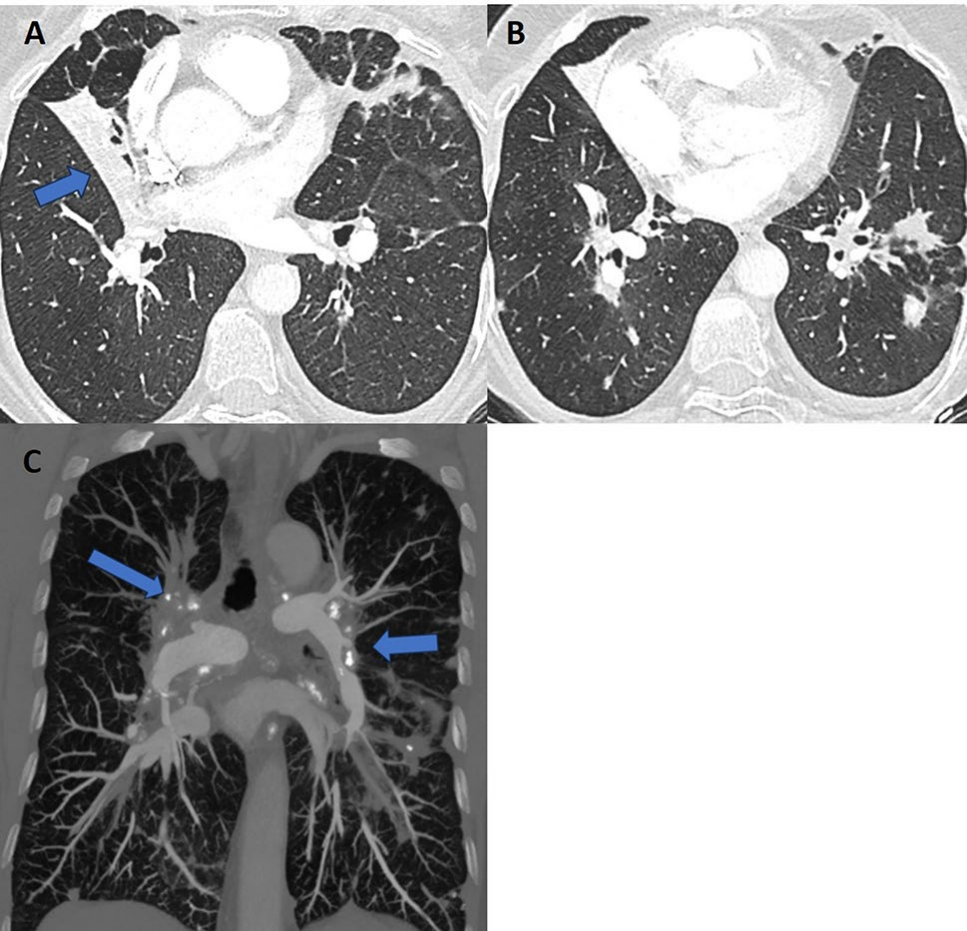
Computed tomography of the chest at the time of pulmonary hypertension diagnosis: (**a**) and (**b**) axial images show irregular diffuse nodular and micronodular lesions forming conglomerates and causing bronchial stenosis, atelectasis of the middle lobe (blue arrow) with no endobronchial lesions and impaired ventilatory pattern of the lingula, areas of ground glass and air trapping; (**c**) coronal MIP reconstruction reveals multiple slightly enlarged hilar and bronchopulmonary lymph nodes with calcifications (blue arrows)

**Fig. 3:**
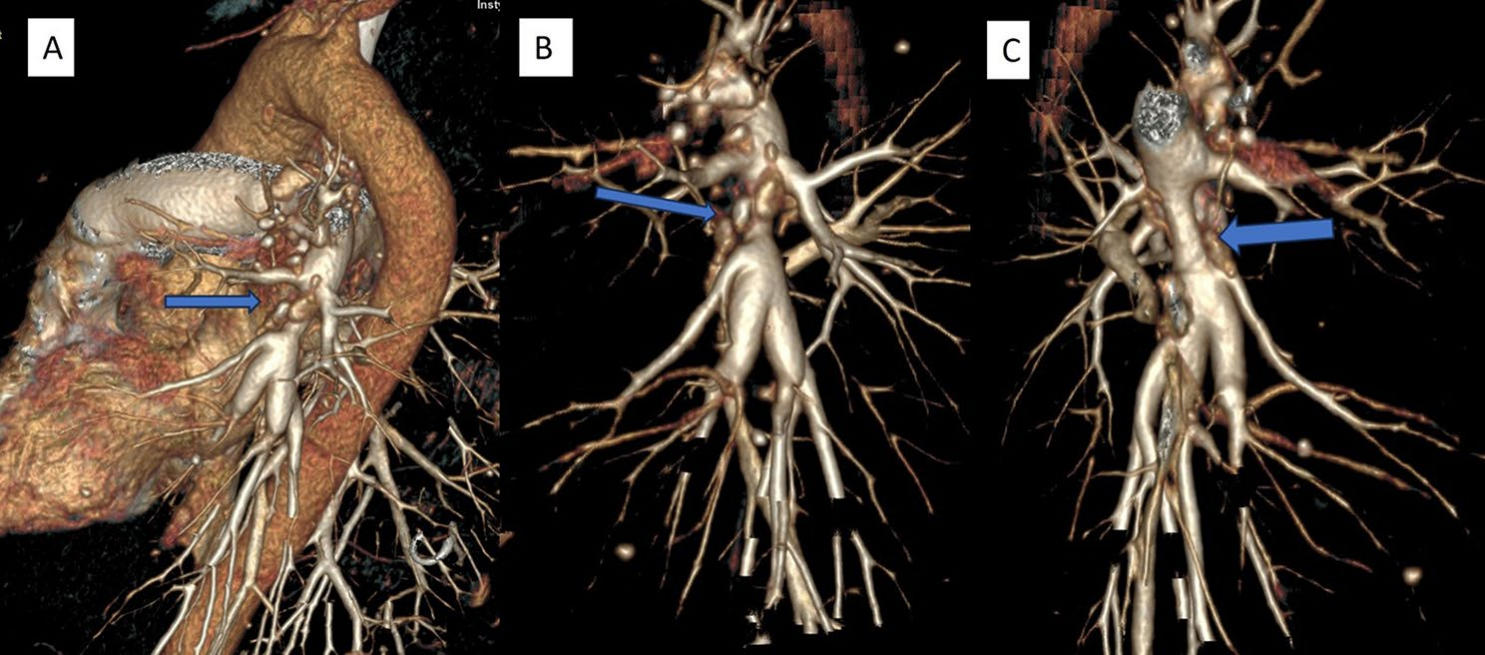
Computed tomography pulmonary angiography, 3D reconstructions precisely show narrowing of the left pulmonary artery and adjacent calcified lymph nodes (blue arrows) (**A**) lateral view (**B**) oblique view, and (**C**) posterior view

CT pulmonary angiography (CTPA) showed no pulmonary embolism. However, it revealed dilatation of the pulmonary artery trunk, right ventricle and atrium, complete occlusion of the right upper lobe artery shortly after leaving the pulmonary trunk, and narrowing of the left lower lobe artery just below the branch of the artery to segment six with post-stenotic dilatation of the arteries of the basal segments of the left lower lobe (Figs. 1, 2 and 3). The walls of the arteries in these sections were not thickened. The occlusion and narrowing of the arteries were caused by adjacent, enlarged lymph nodes with calcifications and fibrous tissue surrounding the vessels.

Oxygen therapy and diuretics were implemented with gradual decrease of lower limbs oedema and dyspnoea. Considering the progression of lymphadenopathy and micronodular lesions in the lungs in a previously untreated sarcoidosis patient, systemic glucocorticoids were introduced (prednisone 40 mg/day). The resolution of respiratory failure and improvement in WHO functional class from IV to III have been achieved. Pulmonary function tests revealed a mild obstructive ventilatory pattern with FEV_1_ of 75% predicted, FVC of 86% predicted, FEV_1_/FVC 67%, total lung capacity of 99% predicted, and a reduced TLCO of 52% predicted. The six-minute walk distance (6MWD) without supplemental oxygen was 340 m without significant desaturation (from 94 to 93%). A follow-up echocardiography showed a decrease in TRV to 4,2 m/s, and sPAP - to 75 mmHg after three weeks of treatment. The right ventricle diameter, in four - chamber view, decreased to 37 mm, right atrium area decreased from 21 cm^2^ to 12 cm^2^ and TAPSE increased to 20 mm.

The patient was reevaluated after three months of corticosteroid therapy, and reported significant relief of symptoms (WHO functional class II/III). Control chest CT showed partial regression of disseminated micronodular lesions and conglomerates, a slight reduction of mediastinal lymph nodes, complete regression of right sided pleural fluid and significant regression of left-sided one. The dose of corticosteroid was reduced and immunosuppressive treatment with methotrexate at an initial dose of 7.5 mg once a week ( reduced due to chronic renal insufficiency) was initiated. Since echocardiography continued to show signs of severe pulmonary hypertension with TRV 4.12 m/s, sPAP 70 mmHg, the patient was referred to a tertiary centre of pulmonary circulation (Department of Pulmonary Circulation, Thromboembolic Diseases and Cardiology, European Health Center, Otwock) to extend the diagnostic evaluation and determine further therapeutic management.

**Fig. 4:**
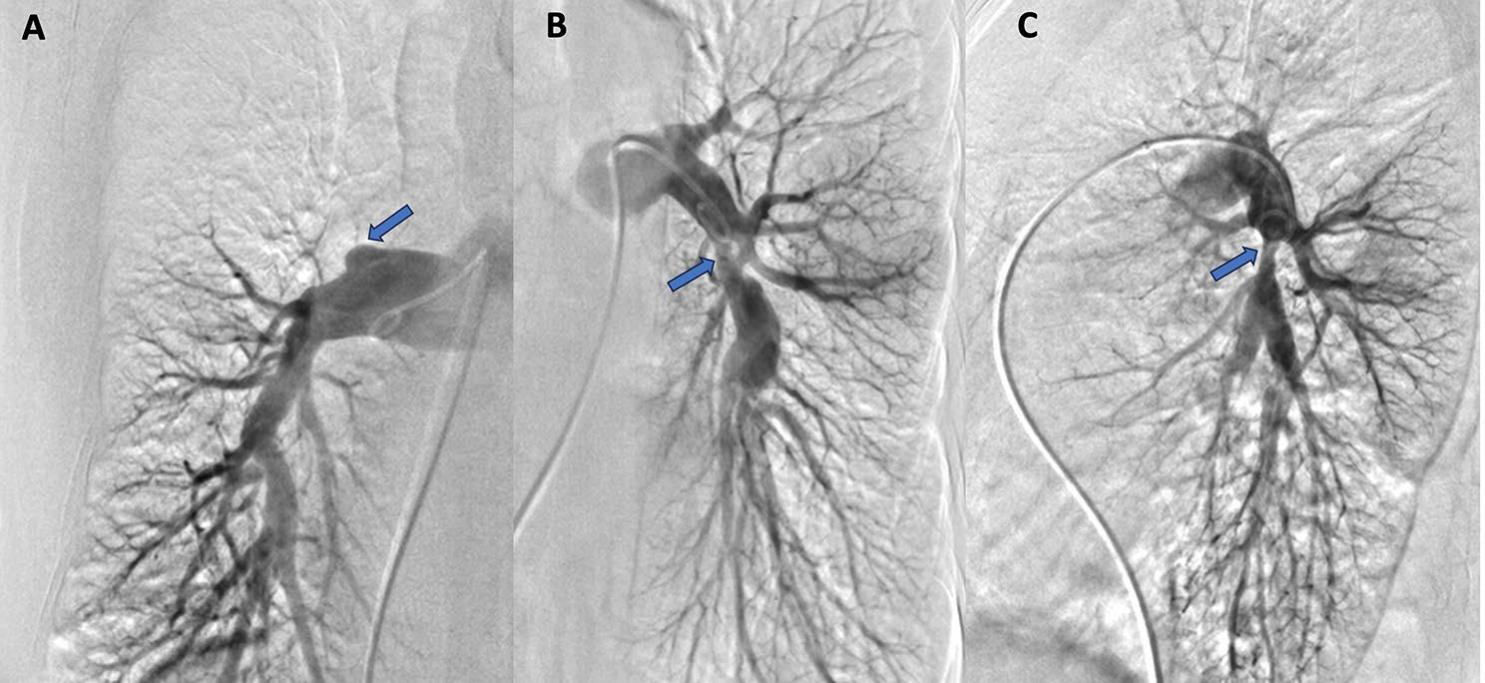
Selective digital subtraction angiography of the pulmonary artery. (**A**) Right pulmonary artery - PA view; (**B**) Left pulmonary artery - PA view; (**C**) Left pulmonary artery - lateral view. Total occlusion and ring-like stenosis marked with arrows

A right heart catheterisation (RHC) followed by pulmonary angiography showed signs of precapillary pulmonary hypertension with mean pulmonary artery pressure (mPAP) of 37 mmHg, normal right atrial pressure of 2 mmHg and pulmonary capillary wedge pressure (PCWP) of 8 mmHg, a decreased cardiac index of 2.06 l/min/m^2^, and a significantly increased pulmonary vascular resistance (PVR) of 8.92 Wood Units (Table 1). Pulmonary angiography revealed occlusion of the right upper lobe artery and significant concentric stenosis of the left lower lobe artery just after the artery branched into segment six, with post-stenotic widening and similar narrowing of the artery to segment nine (Fig. 4). No chronic thromboembolic lesions suggestive of chronic thromboembolic pulmonary hypertension (CTPEH) were found. The patient was qualified for balloon pulmonary angioplasty (BPA) preceded by intravascular imaging with intravascular ultrasound (IVUS).

**Table 1:** Right heart catheterization (RHC) data before and after the balloon pulmonary angioplasty procedures

Parameter	RHC baseline (before the first BPA procedure)	RHC 5 months after baseline (before the second BPA procedure)	RHC 9 months after baseline (before the stent implementation)	RHC 5 months after the stent implementation
mPAP, mmHg	37	32	29	41
RAP, mmHg	2	4	6	3
PCWP, mmHg	8	8	11	8
CI, l/min/m^2^	2.06	2.03	2.11	2.33
PVR, WU	8.92	7.23	5.19	8.53

Abbreviations: BPA, balloon pulmonary angioplasty; RHC, right heart catheterisation; mPAP, mean pulmonary artery pressure; RAP, right atrial pressure; PCWP, pulmonary capillary wedge pressure; CI, Cardiac index; PVR, pulmonary vascular resistance; WU, Wood units

**Fig. 5:**
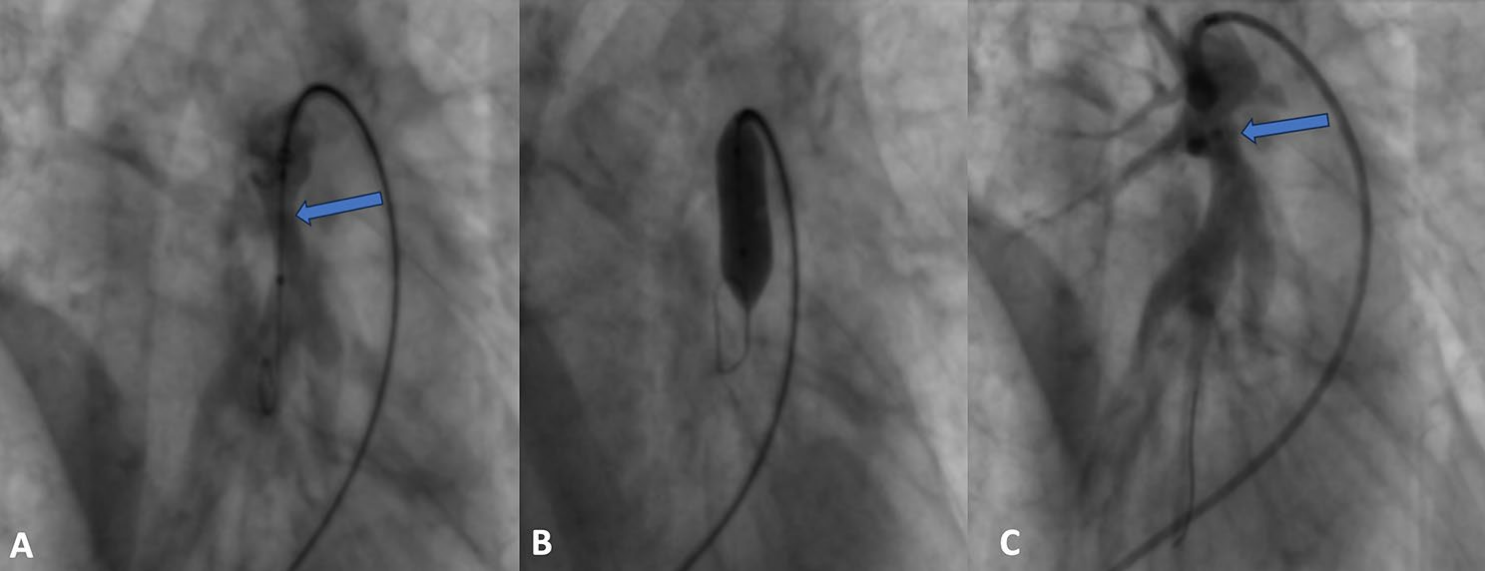
Pulmonary balloon angioplasty of the left lower lobe artery. (**A**) selective angiography before BPA, vessel narrowing marked with arrow; (**B**) BPA with 10 mm angioplasty balloon catheter; (**C**) status post BPA with a widened lumen of the pulmonary artery (arrow)

Two lesions of the type of post-inflammatory stenosis were treated – one in the artery to the left segment nine and one in the left lower lobe artery, with initially good angiographic effect and reduction of mean PAP from 37mmHg to 30 mmHg (Fig. 5). No complications were observed. Due to BPA procedure, anticoagulant treatment with apixaban was initiated.

The repeated BPA procedures were performed 5 months later, and five lesions were treated: three ones in the artery to the left segment nine and their sub-segmental branches and two - in the sub-segmental branches of the artery to the right segment eight. An attempt to obtain the recanalization of the right upper-lobe artery was unsuccessful. Two months after the BPA procedures and one year after the introduction of immunosuppressive treatment, the patient was reassessed in our department. The clinical symptoms were stable, with WHO functional class II/III. The 6MWD without supplemental oxygen was 410 m with desaturation from 97 to 92%. TTE revealed pressure overload of the right ventricle with TRV 4.69 m/s; sPAP 91 mmHg. As the BPA procedure was ineffective, stent implantation into the left lower-lobe artery was performed, resulting in a reduction in pulmonary artery pressure (TRV 4 m/s; sPAP 70 mmHg). Unfortunately, a follow-up RHC five months after stent implementation showed deterioration of hemodynamic parameters (Table 1). After multidisciplinary discussion and presenting the patient with the benefits and risks of such management, sildenafil (PAH-dedicated therapy) was included as an “off-label” treatment with initially good tolerance.

## Discussion

The most common cause of SAPH development is lung fibrosis with the associated destruction of the vascular bed, and/or chronic alveolar hypoxia. According to registry data and a large single-center cohort, 66–72% of SAPH patients were diagnosed with lung fibrosis on chest X-ray (Scadding stage 4) [11, 16, 24]. Given that the median time between the diagnosis of sarcoidosis and SAPH is about 15 to 17 years, pulmonary hypertension in sarcoidosis patients is considered a complication of the advanced disease stage [11, 25]. The predominant pathophysiological mechanism of PH on such occasion resembles group 3 PH associated with chronic lung diseases [12, 26]. However, some differences have been observed between SAPH and PH due to other interstitial lung diseases (ILDs), such as the presence of higher PAP in sarcoidosis patients than in patients with idiopathic pulmonary fibrosis or other fibrotic ILDs [27, 28]. Moreover, no correlation could be found between haemodynamic parameters from RHC and spirometry or plethysmography results in SAPH patients [11, 16, 29, 30]. According to the data from international, multicentre registries, including the international Registry for Sarcoidosis Associated Pulmonary Hypertension (ReSAPH) and the French Pulmonary Hypertension Registry, approximately 30% of SAPH patients had no significant fibrosis on chest imaging. Moreover, pulmonary hypertension was observed in 2 to 5% of patients with stage I sarcoidosis [11, 25]. These data suggested the involvement of other mechanisms in the development of pre-capillary pulmonary hypertension in sarcoidosis patients. Direct involvement of pulmonary vessels, extrinsic compression of pulmonary vessels, and pulmonary embolism have been suggested in the current literature [12, 13, 20, 31–33]. Histologically, pulmonary vascular involvement in sarcoidosis patients is very common and has been reported, depending on the type of specimen for pathological examination, from 100% - in autopsy studies to 41% - in lung biopsy specimens [34, 35]. Granulomatous pulmonary angiitis was found in both arterial and venous pulmonary vasculature of all calibres, and was reported in all 40 autopsy cases of sarcoidosis as well as in 80% of explanted lungs from SAPH patients [29, 35].

Another form of intrinsic vascular involvement is microangiopathy, which refers to the alterations of pulmonary capillary vessels. These lesions were revealed in 35% of transbronchial biopsy samples from sarcoidosis patients, and in all explanted lungs of SAPH patients [29, 34]. As granulomatous inflammation primarily affects the pulmonary veins, causing their occlusion and destruction, the clinical picture may resemble pulmonary venoocclusive disease [21].

In situ thrombosis and venous thromboembolic disease (VTE) are other mechanisms of intrinsic pulmonary artery obstruction described in sarcoidosis patients [13, 21, 32, 36]. VTE is more frequent in sarcoidosis compared to the general population, thus chronic thromboembolic pulmonary hypertension (CTEPH) as the cause of SAPH has to be also considered [23, 32, 33, 37, 38]. Several recently published studies have reported a twofold to fourfold increased risk of VTE in sarcoidosis patients compared to controls [36, 38]. The underlying cause of the association between sarcoidosis and VTE has not been firmly established. Some authors have hypothesized that the chronic inflammatory process, that is associated with sarcoidosis, may predispose to a hypercoagulable state through endothelial cell injury, increased release of cytokines activating the coagulation cascade, upregulation of tissue factor, suppression of fibrinolysis and downregulation of thrombomodulin by tumour necrosis factor-α [32]. Other potential risk factors for VTE include treatment with corticosteroids or the presence of antiphospholipid antibodies, observed in up to 38% of sarcoidosis patients [39, 40].

Extrinsic pulmonary vasculopathy in sarcoidosis may be caused by markedly enlarged mediastinal and/or hilar lymph nodes. However, compression has to affect multiple vessels to lead to severe pulmonary hypertension. In such circumstances, the immunosuppressive therapy may diminish lymph nodes’ diameter, resulting in the decrease in vascular compression. Extrinsic compression due to fibrotic lung lesions or fibrosing mediastinitis are much more difficult to treat. According to the suggested management approach to patients with SAPH caused by external pulmonary vascular compression, the evaluation of disease activity on PET/CT scan may be important. In case of active disease, corticosteroid and immunosuppressive treatment, are recommended [31, 41]. Otherwise, balloon pulmonary angioplasty, which is currently recommended for the therapy of inoperable chronic thromboembolic pulmonary hypertension, or pulmonary artery stenting performed in PH expert centre may be considered [23, 42–44]. In case of failure or insufficient response to treatment and deterioration of lung function, early referral for lung or lung and heart transplantation is recommended.

The most likely cause of pulmonary hypertension in the presented patient was an extrinsic vascular compression by enlarged lymph nodes with calcifications and by surrounding fibrotic lung tissue. This mechanism has been reported to affect from 5 to 21.4% of patients with SAPH [11, 13, 29]. Mathijssen et al. found this phenotype in six their patients (15%), in whom pulmonary vascular compression was caused by fibrosis or calcified lymph nodes [13]. One of these patients was diagnosed with fibrosing mediastinitis, defined as progressive proliferation of dense infiltrative fibrous tissue in the mediastinum replaces normal mediastinal fat. The predominant cause of pulmonary artery stenosis in the case we described was compression by adjacent enlarged lymph nodes with calcifications. In addition, complete occlusion of the right upper lobe artery shortly after leaving the pulmonary trunk was caused by fibrous tissue surrounding the vessels and by enlarged lymph nodes. Given the radiological pattern on contrast-enhanced computed tomography, it would be necessary to consider the diagnosis of fibrosing mediastinitis in our patient. This rare benign but potentially life-threating process causing chronic pulmonary artery and /or vein occlusion results in pulmonary hypertension and right-sided hart failure. The presence on a contrast-enhanced CT scan of signs of pulmonary hypertension and atelectasis, or both, and a refractory pleural effusion should suggest to the physician the diagnosis of pulmonary hypertension associated with mediastinal fibrosis [45, 46].

Additionally, granulomatous vasculitis and secondary post-inflammatory narrowing of the pulmonary arteries were considered in the presented patient. The implementation of typical heart failure treatment and the addition of a systemic corticosteroid combined with immunosuppressant resulted in a fairly rapid improvement in our patient’s clinical status, a decrease in sPAP on echocardiography, a resolution of respiratory failure, and an improvement of functional status. The use of BPA procedures had only a transient effect on hemodynamic parameters at RHC, and did not significantly affect hemodynamic and functional status, that might indicate that the restenosis occurred. As the BPA procedure was ineffective, stent implantation into the left lower-lobe artery was performed, resulting in a reduction in pulmonary artery pressure on echocardiography. In a prospective study of 72 patients with SAPH, Liu et al. reported significant pulmonary artery stenosis in 8 (11%) patients. All of them underwent successful stenting with reduction in pulmonary artery pressure [47]. In addition, a recent meta-analysis of the available literature published by DaSilva et al. showed that pulmonary angioplasty with or without stenting may lead to significant improvement in 6MWD in SAPH patients [48]. In contrast, in our patient, a follow-up RHC 5 months after stent implementation unfortunately showed deterioration of hemodynamic parameters. After multidisciplinary discussion, PAH-targeted therapy with sildenafil was started, with initially good tolerance of treatment.

SAPH therapy data are scarce, and not all studies found a positive response to this treatment. There is currently no registered drug for the treatment of SAPH. According to the WASOG statement on the diagnosis and management of SAPH, the off-label use of specific PAH therapies should be considered on a case-by-case basis by a multidisciplinary team with a sarcoidosis and a PH expert [19].

The international registry data indicate the long latency period from initial diagnosis of sarcoidosis to development of PH (up to 17 years), suggesting the possibility of delayed diagnosis of SAPH [11, 25]. In the presented case, the delay from the diagnosis to the recognition of PH was 12 years, and the first echocardiography, performed in the highly symptomatic period of disease, indicated severe pulmonary hypertension, which was subsequently confirmed by an RHC. The delay in the diagnosis of pulmonary hypertension and the lack of prior immunosuppressive therapy may have affected the efficacy of our treatment. Thus, it would be advisable to raise doctors’ awareness of the need to consider pulmonary hypertension as a cause of persistent dyspnoea in sarcoidosis patients. Further clinical trials are also needed to determine the importance of different forms of therapy for this complication.

It is therefore one of the most difficult challenges in the management of SAPH patients to establish an early, appropriate diagnosis. There are currently no adequately validated screening methods, and data regarding the consequences of SAPH screening remain unknown. In the recently published WASOG statement the task force suggests transthoracic echocardiography to determine the probability of pulmonary hypertension in sarcoidosis patients when one or more of the following features are present: persistent dyspnoea despite aggressive anti-inflammatory treatment, clinical signs of right ventricular failure, a reduced distance of less than 300 m or greater than 5% desaturation at six-minute walk test (6MWT), greater than 20% decrease in 6MWD without modification in pulmonary function tests, high level of brain natriuretic peptides, pulmonary fibrosis of more than 20% of the lung parenchyma, features of PH on CT scan, right ventricular dysfunction or suspicion of PH on magnetic resonance imaging, worsening of NYHA functional class or a greater than 15% decrease of the TLCO when there was no significant change in lung volumes [19]. Echocardiography should be used to determine who should undergo RHC, which remains the gold standard for diagnosing PH.

The committee members suggest performing RHC if there is a high probability of PH or intermediate probability of PH on echocardiography in patients with FVC less than 50% of the predicted value. In other cases, the decision to perform this invasive procedure should be made after multidisciplinary discussion on a case-by-case basis [19].

## Conclusions

SAPH is a rare condition, but significantly affects morbidity and mortality. The mechanisms leading to the development of pulmonary hypertension in patients with sarcoidosis are multifactorial and complex, hence, according to the WHO classification, it belongs to group 5. Although the most common cause of pulmonary hypertension in sarcoidosis patients is advanced lung disease with vascular bed damage and alveolar hypoxia, a substantial proportion of patients (up to 30%) do not have significant fibrosis on chest imaging. In such cases, the development of pulmonary hypertension may be due to lesions directly affecting the pulmonary vasculature, such as granulomatous angiitis, microangiopathy, chronic thromboembolism or external compression of vessels by enlarged lymph nodes and pulmonary fibrosis. Identifying the underlying phenotype of SAPH is important to determine the appropriate therapeutic approach, such as immunosuppressive therapy and/or PAH-targeted treatment. Balloon pulmonary angioplasty with or without stenting might be an option in case of pulmonary artery stenosis.

## Data Availability

The datasets used and analysed in this paper are available from the corresponding author on reasonable request.

## Abbreviations

SAPH: sarcoidosis-associated pulmonary hypertension
PH: pulmonary hypertension
ESC: European Society of Cardiology
ERS: European Respiratory Society
PAH: pulmonary arterial hypertension
CTEPH: chronic thromboembolic pulmonary hypertension
FEV_1_: forced expiratory volume in one second
FVC: forced vital capacity
TLCO: lung transfer factor for carbon monoxide
CT: computed tomography
CTPA: computed tomography pulmonary angiography
WHO: World Health Organization
WBC: white blood cell
NT-pro BNP: N-terminal pro-B-type natriuretic peptide
6MWD: six-minute walk distance
6MWT: six-minute walk test
RHC: right heart catheterization
mPAP: mean pulmonary artery pressure
PCWP: pulmonary capillary wedge pressure
PVR: pulmonary vascular resistance
IVUS: balloon pulmonary angioplasty
BPA: intravascular ultrasound
TTE: transthoracic echocardiography
ILDs: interstitial lung diseases
TVPG: tricuspid valve pressure gradient
VTE: venous thromboembolic disease
PET/CT: positron emission tomography/computed tomography
